# Supplementary material for: Exploring the Rumen and Cecum Microbial Community from Fetus to Adulthood in Goat
Source: Animals (Basel). 2020 Sep 11;10(9):1639. doi: 10.3390/ani10091639 (PMC7552217; doi:10.3390/ani10091639)
Supplement: Supplementary file 1 [file animals-10-01639-s001.zip › Supplementary File(s)/Table S1.docx]

**Table S1 The profile of goats.** All goats were slaughtered in two commercial abattoirs. The date from 23 to 25 Dcember 2017 was on the same commercial abattoir, and from 2 to 3 January 2018 was on another commercial abattoir.

| Stage | | Number | Number of embryos | Date of slaughter | collection of samples |
| --- | --- | --- | --- | --- | --- |
| Pregnant goats | 90 days | 3 | 9 | two on 23 Dcember 2017, and one on 2 January 2018 | pregnant goats’rumen content (n=9), cecum content (n=9), umbilical cord blood (n=9), amniotic fluid (n=3) and their fetus’ rumen fluid (n=22) and cecum content (n=22) |
|  | 100 days | 3 | 6 | one on 24 Dcember 2017, and two on 2 January 2018 |  |
|  | 120 days | 3 | 7 | all on 3 January 2018 |  |
| Lambs | Newborn | 4 | - | two on 23 Dcember 2017, and two on 3 January 2018 | rumen fluid (n=16) and cecum content (n=16) |
|  | One day old | 4 | - | two on 24 Dcember 2017, and two on 3 January 2018 |  |
|  | Three months old | 3 | - | one on 23 Dcember 2017, one on 25 Dcember 2017 and one on 3 January 2018 |  |
|  | Six months old | 5 | - | two on 23 Dcember 2017, one on 25 Dcember 2017 and two on 3 January 2018 |  |
