# Supplementary material for: Exploring the Rumen and Cecum Microbial Community from Fetus to Adulthood in Goat
Source: Animals (Basel). 2020 Sep 11;10(9):1639. doi: 10.3390/ani10091639 (PMC7552217; doi:10.3390/ani10091639)
Supplement: Supplementary file 1 [file animals-10-01639-s001.zip › Supplementary File(s)/Table S11.docx]

**Table S11 Analysis of similarity (ANOSIM) of bacterial microbiota among all groups.** This analysis provides a way to statistically test whether there is a significant difference between two or more groups of samples, by generating a similarity value (R-value) between 0 and 1 using the Bray-Curtis index. R-values closer to 0 represent groups that do not significantly differ, while values closer to 1 represent a highly different community composition. Upper side triangle is the R-value; lower side triangle is the *P* value between the groups according to the ANOSIM values.

|  | FR | NBR | LR1d | LR3m | LR6m | GR | FC | NBC | LC1d | LC3m | LC6m | GC | UCB | AF |
| --- | --- | --- | --- | --- | --- | --- | --- | --- | --- | --- | --- | --- | --- | --- |
| FR | - | 0.524 | 0.847 | 0.844 | 0.954 | 0.867 | 0.053 | 0.014 | 1.000 | 0.885 | 0.886 | 0.801 | 0.009 | 0.304 |
| NBR | 0.004 | - | 0.542 | 1.000 | 1.000 | 1.000 | 0.506 | 0.375 | 0.969 | 1.000 | 1.000 | 0.999 | 0.791 | 1.000 |
| LR1d | 0.001 | 0.063 | - | 0.704 | 0.781 | 0.807 | 0.951 | 0.604 | 0.510 | 0.870 | 0.963 | 0.979 | 0.835 | 0.667 |
| LR3m | 0.001 | 0.037 | 0.066 | - | 0.456 | 0.411 | 0.968 | 1.000 | 1.000 | 1.000 | 1.000 | 1.000 | 0.820 | 1.000 |
| LR6m | 0.001 | 0.008 | 0.008 | 0.092 | - | 0.707 | 0.982 | 1.000 | 1.000 | 1.000 | 1.000 | 1.000 | 0.958 | 1.000 |
| GR | 0.001 | 0.002 | 0.004 | 0.066 | 0.002 | - | 0.978 | 1.000 | 1.000 | 1.000 | 1.000 | 1.000 | 0.858 | 1.000 |
| FC | 0.076 | 0.002 | 0.001 | 0.001 | 0.001 | 0.001 | - | 0.033 | 1.000 | 0.972 | 0.978 | 0.967 | 0.038 | 0.314 |
| NBC | 0.411 | 0.031 | 0.032 | 0.038 | 0.012 | 0.003 | 0.370 | - | 1.000 | 1.000 | 1.000 | 1.000 | 1.000 | 1.000 |
| LC1d | 0.001 | 0.036 | 0.025 | 0.032 | 0.007 | 0.004 | 0.001 | 0.024 | - | 1.000 | 1.000 | 1.000 | 1.000 | 1.000 |
| LC3m | 0.001 | 0.034 | 0.054 | 0.096 | 0.015 | 0.004 | 0.002 | 0.024 | 0.026 | - | 0.580 | 0.755 | 1.000 | 1.000 |
| LC6m | 0.001 | 0.010 | 0.006 | 0.013 | 0.007 | 0.001 | 0.001 | 0.007 | 0.007 | 0.056 | - | 0.548 | 1.000 | 1.000 |
| GC | 0.001 | 0.003 | 0.001 | 0.004 | 0.001 | 0.001 | 0.001 | 0.002 | 0.003 | 0.011 | 0.001 | - | 1.000 | 1.000 |
| UCB | 0.412 | 0.001 | 0.002 | 0.008 | 0.002 | 0.001 | 0.231 | 0.041 | 0.004 | 0.008 | 0.002 | 0.001 | - | 0.444 |
| AF | 0.030 | 0.033 | 0.047 | 0.099 | 0.018 | 0.008 | 0.007 | 0.027 | 0.032 | 0.099 | 0.018 | 0.004 | 0.004 | - |
