# Supplementary material for: Exploring the Rumen and Cecum Microbial Community from Fetus to Adulthood in Goat
Source: Animals (Basel). 2020 Sep 11;10(9):1639. doi: 10.3390/ani10091639 (PMC7552217; doi:10.3390/ani10091639)
Supplement: Supplementary file 1 [file animals-10-01639-s001.zip › Supplementary File(s)/Table S2.docx]

**Table S2 Abundance of microbiota in negative controls, fetus rumen (FR) and cecum content (FC) in genus level.** 1 the relative abundance of microbiota in FR or FC groups was >1%, while in negative controls was <1%.

| **Genus** | Relative abundance (%) | | |
| --- | --- | --- | --- |
|  | NC | FR | FC |
| Unclassified_Burkholderiales | 13.12 | 19.88 | 18.17 |
| Unclassified_Comamonadaceae | 8.75 | 32.08 | 29.83 |
| Ralstonia^1^ | 0.03 | 0.01 | 15.61 |
| Prevotella^1^ | 0.65 | 3.95 | 2.16 |
| Unclassified_Clostridiales | 2.28 | 2.89 | 2.26 |
| Acinetobacter | 9.42 | 10.96 | 2.77 |
| Ochrobactrum | 8.1 | 3.87 | 1.85 |
| Unclassified_S24-7 | 4.42 | 0.44 | 0.15 |
| Sphingomonas | 4.28 | 2.67 | 0.18 |
| Deinococcus | 4.24 | 2.47 | 0.04 |
| Methylobacterium | 3.04 | 2.12 | 0.07 |
| Chryseobacterium | 1.42 | 1.98 | 0.08 |
| Paucibacter | 1.63 | 1.01 | 1.86 |
| Thermus | 2.58 | 0.8 | 0.2 |
| Unclassified_Caulobacteraceae | 2.31 | 1.44 | 0.15 |
| Lactobacillus | 1.06 | 1.31 | 1 |
| Unclassified_Methylobacteriaceae | 2.6 | 1.79 | 0.05 |
| Methylibium1 | 0.7 | 0.76 | 2.63 |
| Enterobacteriaceae;g__Serratia | 1.44 | 0.99 | 0.13 |
| Unclassified_Sphingomonadaceae | 1.53 | 1.1 | 0.03 |
| Leadbetterella | 2.97 | 0 | 0 |
| Unclassified_Ruminococcaceae^1^ | 0 | 3.67% | 1.85% |
| Unclassified_Bacteroidales1 | 0.02 | 2.16% | 1.15% |
