# Supplementary material for: Exploring the Rumen and Cecum Microbial Community from Fetus to Adulthood in Goat
Source: Animals (Basel). 2020 Sep 11;10(9):1639. doi: 10.3390/ani10091639 (PMC7552217; doi:10.3390/ani10091639)
Supplement: Supplementary file 1 [file animals-10-01639-s001.zip › Supplementary File(s)/Table S3.docx]

**Table S3 The data rejection on the animal microbiota samples (Related to Figure 2B)**

| Group | ID | Rejected | Accepted |
| --- | --- | --- | --- |
| FC | 1 | 27395 | 11852 |
| FC | 2 | 27306 | 14881 |
| FC | 3 | 24781 | 17857 |
| FC | 4 | 31760 | 8477 |
| FC | 5 | 24707 | 21188 |
| FC | 6 | 39960 | 13414 |
| FC | 7 | 24856 | 13266 |
| FC | 8 | 14985 | 14491 |
| FC | 9 | 24617 | 14339 |
| FC | 10 | 25783 | 7221 |
| FC | 11 | 24194 | 9249 |
| FC | 12 | 18102 | 11589 |
| FC | 13 | 12153 | 19160 |
| FC | 14 | 25319 | 9693 |
| FC | 15 | 24038 | 11052 |
| FC | 16 | 19762 | 12299 |
| FC | 17 | 26517 | 12583 |
| FC | 18 | 23391 | 11434 |
| FC | 19 | 26134 | 11751 |
| FC | 20 | 27375 | 10012 |
| FC | 21 | 38559 | 19541 |
| FC | 22 | 16225 | 13763 |
| FR | 1 | 20034 | 21062 |
| FR | 2 | 23743 | 19344 |
| FR | 3 | 20457 | 25783 |
| FR | 4 | 23948 | 16135 |
| FR | 5 | 28348 | 18592 |
| FR | 6 | 28199 | 19246 |
| FR | 7 | 28483 | 6731 |
| FR | 8 | 18562 | 20612 |
| FR | 9 | 11262 | 14068 |
| FR | 10 | 11572 | 18668 |
| FR | 11 | 28394 | 14034 |
| FR | 12 | 9285 | 14057 |
| FR | 13 | 10709 | 17054 |
| FR | 14 | 12905 | 16820 |
| FR | 15 | 38098 | 9412 |
| FR | 16 | 25014 | 18282 |
| FR | 17 | 34485 | 7667 |
| FR | 18 | 22284 | 15911 |
| FR | 19 | 29757 | 9937 |
| FR | 20 | 31887 | 9670 |
| FR | 21 | 30190 | 16621 |
| FR | 22 | 26463 | 16291 |
| GC | 1 | 11985 | 24006 |
| GC | 2 | 17636 | 10891 |
| GC | 3 | 8077 | 9974 |
| GC | 4 | 7670 | 25187 |
| GC | 5 | 11061 | 14989 |
| GC | 6 | 5130 | 12194 |
| GC | 7 | 2528 | 7291 |
| GC | 8 | 2663 | 8429 |
| GC | 9 | 2235 | 8027 |
| GR | 1 | 796 | 17665 |
| GR | 2 | 1082 | 25105 |
| GR | 3 | 9041 | 33533 |
| GR | 4 | 1591 | 27798 |
| GR | 5 | 556 | 25931 |
| GR | 6 | 1095 | 36852 |
| GR | 7 | 809 | 8689 |
| GR | 8 | 809 | 11485 |
| GR | 9 | 618 | 11786 |
| LC1d | 1 | 5827 | 51611 |
| LC1d | 2 | 28445 | 39209 |
| LC1d | 3 | 3731 | 22313 |
| LC1d | 4 | 15479 | 24829 |
| LC3m | 1 | 9132 | 20808 |
| LC3m | 2 | 4362 | 20525 |
| LC3m | 3 | 6136 | 16105 |
| LC6m | 1 | 2751 | 26705 |
| LC6m | 2 | 7438 | 15689 |
| LC6m | 3 | 6457 | 16752 |
| LC6m | 4 | 2032 | 22361 |
| LC6m | 5 | 6769 | 24802 |
| LR1d | 1 | 357 | 34978 |
| LR1d | 2 | 35337 | 27794 |
| LR1d | 3 | 399 | 29643 |
| LR1d | 4 | 1331 | 38997 |
| LR3m | 1 | 1613 | 25690 |
| LR3m | 2 | 527 | 40062 |
| LR3m | 3 | 1046 | 28837 |
| LR6m | 1 | 4491 | 45736 |
| LR6m | 2 | 818 | 25567 |
| LR6m | 3 | 271 | 36156 |
| LR6m | 4 | 853 | 31380 |
| LR6m | 5 | 671 | 33082 |
| NBC | 1 | 30092 | 25160 |
| NBC | 2 | 25899 | 31704 |
| NBC | 3 | 21605 | 14258 |
| NBC | 4 | 7397 | 18449 |
| NBR | 1 | 1432 | 43062 |
| NBR | 2 | 3165 | 35583 |
| NBR | 3 | 13270 | 20358 |
| NBR | 4 | 10821 | 21744 |
| UCB | 1 | 10505 | 19086 |
| UCB | 2 | 18250 | 11872 |
| UCB | 3 | 9587 | 15544 |
| UCB | 4 | 7495 | 16953 |
| UCB | 5 | 36034 | 13249 |
| UCB | 6 | 19860 | 13313 |
| UCB | 7 | 11638 | 33601 |
| UCB | 8 | 30479 | 19205 |
| UCB | 9 | 24477 | 12391 |
