# Supplementary material for: Exploring the Rumen and Cecum Microbial Community from Fetus to Adulthood in Goat
Source: Animals (Basel). 2020 Sep 11;10(9):1639. doi: 10.3390/ani10091639 (PMC7552217; doi:10.3390/ani10091639)
Supplement: Supplementary file 1 [file animals-10-01639-s001.zip › Supplementary File(s)/Table S6.docx]

**Table S6A Alpha diversity of bacterial community according to different stages in rumen and cecum.** Mean values with different letters within the same column differ significantly (*P* < 0.05). The same as below in Table S6B. SEM = standard error of mean.

| **Regions** | **Stages** | **Chao** | **Ace** | **Shannon** | **Simpson** |
| --- | --- | --- | --- | --- | --- |
| Rumen | 90 days fetus | 523.45±67.74b | 550.51±71.40b | 4.68±0.53d | 0.78±0.04c |
|  | 100 days fetus | 757.75±48.33a | 808.58±53.46a | 6.04±0.16b | 0.90±0.01ab |
|  | 120 days fetus | 697.43±57.76a | 747.90±62.78a | 5.80±0.38bc | 0.88±0.04b |
|  | New born | 382.32±93.04bc | 420.10±105.30bc | 4.61±0.57cd | 0.85±0.05bc |
|  | 1 day | 282.81±135.85c | 294.30±143.40c | 4.84±0.85bcd | 0.86±0.07bc |
|  | 3 months | 464.65±34.01bc | 477.10±34.64bc | 6.48±0.25ab | 0.96±0.02ab |
|  | 6 montgs | 349.73±56.63bc | 358.40±58.58bc | 6.15±0.36b | 0.96±0.01ab |
|  | Pregnancy goat | 763.22±42.93a | 787.00±44.82a | 7.80±0.22a | 0.98±0.01a |
|  | *P* | <0.0001 | <0.0001 | <0.0001 | 0.0012 |
| Cecum | 90 days fetus | 617.57±57.97b | 653.32±61.90b | 5.12±0.24b | 0.84±0.01c |
|  | 100 days fetus | 577.18±86.25b | 739.39±111.05ab | 4.91±0.49b | 0.84±0.04c |
|  | 120 days fetus | 360.57±19.59c | 393.16±24.82cd | 3.70±0.35c | 0.74±0.05d |
|  | New born | 540.07±75.77b | 577.40±78.54bc | 5.41±0.67b | 0.87±0.07bc |
|  | 1 day | 170.67±7.80d | 174.16±8.18c | 4.39±0.45bc | 0.87±0.04bc |
|  | 3 months | 591.16±34.96b | 611.14±38.14bc | 7.30±0.03a | 0.98±0.00ab |
|  | 6 montgs | 648.34±76.56b | 674.92±79.45ab | 7.43±0.29a | 0.98±0.00a |
|  | Pregnancy goat | 899.54±33.77a | 853.92±63.06a | 8.09±0.10a | 0.99±0.00a |
|  | *P* | <0.0001 | <0.0001 | <0.0001 | <0.0001 |
|  |  |  |  |  |  |
|  |  |  |  |  |  |
| **Table S6B Alpha diversity of the bacterial community according to different regions at a given stage.** | | | | | |
| **Stages** | **Regions** | **Chao** | **Ace** | **Shannon** | **Simpson** |
| 90 days fetus | Rumen | 523.45±67.74 | 550.51±71.40 | 4.68±0.53 | 0.78±0.04 |
|  | Cecum | 617.57±57.97 | 653.32±61.90 | 5.12±0.24 | 0.84±0.01 |
|  | *P* | 0.3068 | 0.2927 | 0.4531 | 0.2476 |
| 100 days fetus | Rumen | 757.75±48.33 | 808.58±53.46 | 6.04±0.16 | 0.90±0.01 |
|  | Cecum | 577.18±86.25 | 739.39±111.05 | 4.91±0.49 | 0.84±0.04 |
|  | *P* | 0.0977 | 0.5846 | 0.0525 | 0.1314 |
| 120 days fetus | Rumen | 697.43±57.76a | 747.90±62.78a | 5.80±0.38a | 0.88±0.04 |
|  | Cecum | 360.57±19.59b | 393.16±24.82b | 3.70±0.35b | 0.74±0.05 |
|  | *P* | <0.0001 | 0.0002 | 0.0016 | 0.0584 |
| New born | Rumen | 382.32±93.04 | 420.10±105.30 | 4.61±0.57 | 0.85±0.05 |
|  | Cecum | 540.07±75.77 | 577.40±78.54 | 5.41±0.67 | 0.87±0.07 |
|  | *P* | 0.2366 | 0.2761 | 0.3977 | 0.8426 |
| 1 day | Rumen | 282.81±135.85 | 294.30±143.40 | 4.84±0.85 | 0.86±0.07 |
|  | Cecum | 170.67±7.80 | 174.16±8.18 | 4.39±0.45 | 0.87±0.04 |
|  | *P* | 0.4414 | 0.4349 | 0.6562 | 0.8987 |
| 3 months | Rumen | 464.65±34.01 | 477.10±34.64 | 6.48±0.25b | 0.96±0.02 |
|  | Cecum | 591.16±34.96 | 611.14±38.14 | 7.30±0.03a | 0.98±0.00 |
|  | *P* | 0.0604 | 0.06 | 0.0317 | 0.2717 |
| 6 montgs | Rumen | 349.73±56.63b | 358.40±58.58b | 6.15±0.36b | 0.96±0.01 |
|  | Cecum | 648.34±76.56a | 674.92±79.45a | 7.43±0.29a | 0.98±0.00 |
|  | *P* | 0.0139 | 0.0125 | 0.0239 | 0.051 |
| Pregnancy goat | Rumen | 763.22±42.93b | 787.00±44.82 | 7.80±0.22 | 0.98±0.01 |
|  | Cecum | 899.54±33.77a | 853.92±63.06 | 8.09±0.10 | 0.99±0.00 |
|  | *P* | 0.0239 | 0.4 | 0.2374 | 0.3934 |
