# Supplementary material for: Exploring the Rumen and Cecum Microbial Community from Fetus to Adulthood in Goat
Source: Animals (Basel). 2020 Sep 11;10(9):1639. doi: 10.3390/ani10091639 (PMC7552217; doi:10.3390/ani10091639)
Supplement: Supplementary file 1 [file animals-10-01639-s001.zip › Supplementary File(s)/Table S7.docx]

**Table S7 Unweighted pair-group method with arithmetic means (UPGMA) of bacterial microbiota among all groups (related to Figure 4B).**

| **Groups** | GR | FR | GC | FC | UCB | LR3m | LC3m | LR1d | LC1d | LR6m | LC6m | NBR | NBC | AF |
| --- | --- | --- | --- | --- | --- | --- | --- | --- | --- | --- | --- | --- | --- | --- |
| GR | 0.00 | 0.77 | 0.82 | 0.84 | 0.69 | 0.47 | 0.86 | 0.67 | 0.94 | 0.61 | 0.85 | 0.87 | 0.79 | 0.80 |
| FR | 0.77 | 0.00 | 0.73 | 0.43 | 0.45 | 0.74 | 0.75 | 0.67 | 0.87 | 0.76 | 0.74 | 0.64 | 0.52 | 0.65 |
| GC | 0.82 | 0.73 | 0.00 | 0.81 | 0.82 | 0.82 | 0.52 | 0.85 | 0.91 | 0.84 | 0.45 | 0.76 | 0.78 | 0.83 |
| FC | 0.84 | 0.43 | 0.81 | 0.00 | 0.46 | 0.81 | 0.79 | 0.73 | 0.85 | 0.81 | 0.79 | 0.63 | 0.56 | 0.63 |
| UCB | 0.69 | 0.45 | 0.82 | 0.46 | 0.00 | 0.67 | 0.82 | 0.66 | 0.88 | 0.73 | 0.81 | 0.72 | 0.58 | 0.63 |
| LR3m | 0.47 | 0.74 | 0.82 | 0.81 | 0.67 | 0.00 | 0.84 | 0.68 | 0.92 | 0.58 | 0.82 | 0.85 | 0.78 | 0.79 |
| LC3m | 0.86 | 0.75 | 0.52 | 0.79 | 0.82 | 0.84 | 0.00 | 0.84 | 0.88 | 0.86 | 0.54 | 0.73 | 0.77 | 0.85 |
| LR1d | 0.67 | 0.67 | 0.85 | 0.73 | 0.66 | 0.68 | 0.84 | 0.00 | 0.80 | 0.66 | 0.84 | 0.74 | 0.68 | 0.75 |
| LC1d | 0.94 | 0.87 | 0.91 | 0.85 | 0.88 | 0.92 | 0.88 | 0.80 | 0.00 | 0.90 | 0.90 | 0.75 | 0.87 | 0.88 |
| LR6m | 0.61 | 0.76 | 0.84 | 0.81 | 0.73 | 0.58 | 0.86 | 0.66 | 0.90 | 0.00 | 0.82 | 0.84 | 0.78 | 0.80 |
| LC6m | 0.85 | 0.74 | 0.45 | 0.79 | 0.81 | 0.82 | 0.54 | 0.84 | 0.90 | 0.82 | 0.00 | 0.74 | 0.76 | 0.83 |
| NBR | 0.87 | 0.64 | 0.76 | 0.63 | 0.72 | 0.85 | 0.73 | 0.74 | 0.75 | 0.84 | 0.74 | 0.00 | 0.60 | 0.73 |
| NBC | 0.79 | 0.52 | 0.78 | 0.56 | 0.58 | 0.78 | 0.77 | 0.68 | 0.87 | 0.78 | 0.76 | 0.60 | 0.00 | 0.68 |
| AF | 0.80 | 0.65 | 0.83 | 0.63 | 0.63 | 0.79 | 0.85 | 0.75 | 0.88 | 0.80 | 0.83 | 0.73 | 0.68 | 0.00 |
