# Supplementary figures and images for: Exploring the Rumen and Cecum Microbial Community from Fetus to Adulthood in Goat
Source: Animals (Basel). 2020 Sep 11;10(9):1639. doi: 10.3390/ani10091639 (PMC7552217; doi:10.3390/ani10091639)

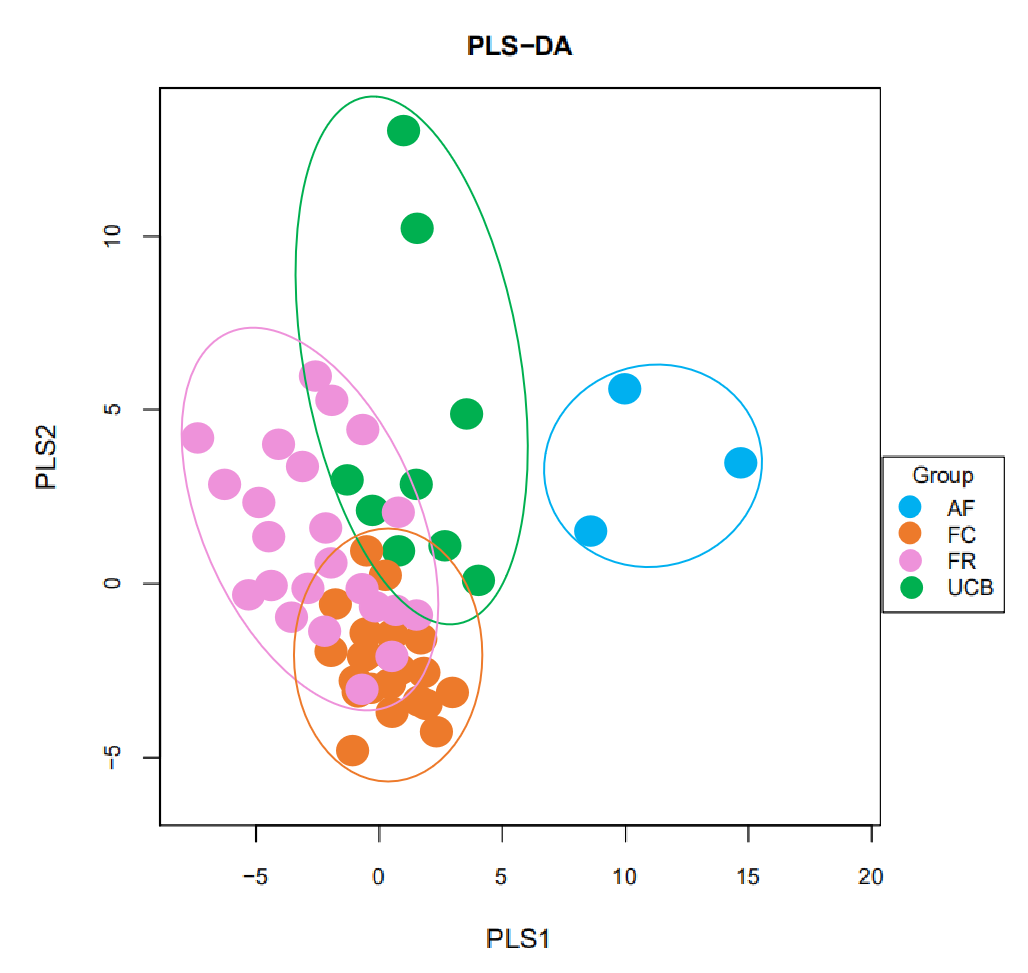

Supplement: Supplementary file 1 [file animals-10-01639-s001.zip › Supplementary File(s)/Figure S1.tif]
